# Clinical and laboratory characteristics of early-onset and delayed-onset lupus nephritis patients: A single-center retrospective study

**DOI:** 10.1007/s00296-024-05579-4

**Published:** 2024-03-28

**Authors:** Joanna Kosałka-Węgiel, Radosław Dziedzic, Andżelika Siwiec-Koźlik, Magdalena Spałkowska, Mamert Milewski, Joanna Żuk-Kuwik, Lech Zaręba, Stanisława Bazan-Socha, Mariusz Korkosz

**Affiliations:** 1 Department of Rheumatology and Immunology, Jagiellonian University Medical College, Jakubowskiego 2, Kraków, 30-688 Poland; 2 Department of Rheumatology, Immunology and Internal Medicine, University Hospital, Jakubowskiego 2, Kraków, 30-688 Poland; 3 Doctoral School of Medical and Health Sciences, Jagiellonian University Medical College, Św. Łazarza 16, Kraków, 31-530 Poland; 4 Department of Dermatology, Jagiellonian University Medical College, Botaniczna 3, Kraków, 31-501 Poland; 5 Department of Hematology, Jagiellonian University Medical College, Jakubowskiego 2, Kraków, 30-688 Poland; 6 College of Natural Sciences, Institute of Computer Science, University of Rzeszów, Pigonia 1, Rzeszów, 35-310 Poland; 7 Department of Internal Medicine, Faculty of Medicine, Jagiellonian University Medical College, Jakubowskiego 2, Kraków, 30-688 Poland

**Keywords:** Systemic lupus erythematosus, Lupus nephritis, Prognostic factors

## Abstract

**Background:**

Lupus nephritis (LN) manifests systemic lupus erythematosus (SLE) and is characterized by various clinical and laboratory features. This study aimed to comprehensively evaluate the characteristics of LN patients according to the time of LN diagnosis: early-onset (LN diagnosed within one year from SLE diagnosis) vs. delayed-onset (LN diagnosed more than one year after SLE diagnosis).

**Methods:**

We conducted a retrospective analysis of medical records from all SLE patients treated at the University Hospital in Kraków, Poland, from 2012 to 2022. We collected data on demographic, clinical, and laboratory characteristics, including histological findings, treatment modalities, and disease outcomes. Statistical analyses were performed to identify factors impacting LN development and prognosis.

**Results:**

Among 331 LN patients, early-onset was diagnosed in 207 (62.54%) and delayed-onset was documented in 122 cases (36.86%). In 2 (0.6%) LN cases, the time of first kidney manifestation in the SLE course was unknown. Delayed-onset LN had a higher female-to-male ratio and younger age at SLE diagnosis. This group was associated with more severe clinical manifestations. In turn, studied subgroups did not differ in internist comorbidities, kidney histopathology, and family history regarding autoimmune diseases. Delayed-onset LN exhibited a higher frequency of anti-dsDNA, anti-Smith, anti-Ro, anti-RNP, and anti-cardiolipin IgG autoantibodies. During a 14-year follow-up period, 16 patients died. Mortality rate and causes of death were comparable in both analyzed subgroups.

**Conclusions:**

More severe clinical manifestations in delayed-onset LN prompt strict monitoring of non-LN SLE patients to diagnose and treat kidney involvement early. Also, recognizing the higher frequency of autoantibodies such as anti-dsDNA or anti-Smith in delayed-onset LN underscores the potential value of autoantibody profiling as a diagnostic and prognostic tool.

## Introduction

Systemic lupus erythematosus (SLE), commonly referred to as lupus, is a multifaceted autoimmune disease that affects multiple organs. SLE is characterized by its diverse clinical manifestations and varied organ involvement. Common symptoms include joint pain and swelling, skin rashes (such as the characteristic butterfly rash), substantial fatigue, photosensitivity, oral ulcers, and fever. Beyond these hallmark symptoms, lupus can affect vital organs, including the kidneys, heart, lungs, and nervous system [1]. The majority of SLE patients experience the onset of the disease between the ages of 16 and 55 years [2]. The pathogenesis of lupus is rooted in the complex interplay between genetic predisposition and environmental triggers. At its core, SLE is typified by a hyperactive immune system that generates autoantibodies directed against self-antigens. These autoantibodies drive a chronic state of inflammation and subsequent tissue damage [3, 4]. Furthermore, this immune dysregulation precipitates cytokine imbalances and pro-inflammatory pathway activation, perpetuating the disease process. The exact triggers for this autoimmune response remain a subject of intense research, with genetic, hormonal, and environmental factors under scrutiny [5].

The kidneys are particularly vulnerable among the various organ systems affected by lupus. Lupus nephritis (LN) is a severe manifestation of SLE characterized by renal inflammation and progressive damage [6]. It usually occurs early during SLE, typically within the initial 3–5 years, and might be evident at the time of the initial diagnosis [7]. If untreated, it progresses to end-stage renal disease [8]. Kidney involvement in lupus often remains asymptomatic until significant damage has occurred, making early detection and intervention crucial. Moreover, LN is associated with increased morbidity and mortality rates, emphasizing the importance of effective management, especially in the early stages [9]. Nevertheless, complete renal remissions are still unfrequent among those receiving standard-of-care treatment [10]. Thus, early diagnosis and prompt therapy are critical to prevent irreversible damage.

The comprehension of clinical and laboratory determinants associated with LN development and its subsequent prognosis assumes pivotal significance in facilitating patient care and management since LN is related to a six-fold increase in mortality compared with the general population [11]. Thus, the study aims to investigate whether SLE patients diagnosed with LN during the first year of SLE duration (early-onset LN) differ in clinical and laboratory characteristics from those diagnosed with LN after more than one year of SLE course (delayed-onset LN). Through this analysis, we aim to illuminate specific aspects of LN, facilitating the development of tailored diagnostic and therapeutic strategies.

## Patients and methods

### Study population

We retrospectively reviewed the medical records of all SLE cases diagnosed and treated at University Hospital, Kraków, Poland, from January 2012 to June 2022. All patients in this study fulfilled the European League Against Rheumatism and the American College of Rheumatology (EULAR/ACR) criteria from 2019 [12]. We collected comprehensive data, including sex, present age, age at the first SLE symptoms, age at the disease diagnosis, time delay between the onset of SLE symptoms and diagnosis, duration of the disease, family history of SLE and other autoimmune diseases, clinical and laboratory SLE manifestations, comorbidities, miscarriages in women, different treatment modalities, and cause and age of death (if applicable). The present age was defined as the age of data completion [13].

The patient cohort was divided into two subgroups according to the time of LN diagnosis. The first group comprised patients who developed LN within one year of the SLE diagnosis (early-onset LN). The second group consisted of those who developed renal involvement more than one year after SLE diagnosis (delayed-onset LN) [14, 15]. The duration of the disease was calculated from the onset of SLE symptoms to the last visit or patient death. The evaluated clinical manifestations included: general symptoms (fever [above 38^o^C with the exclusion of infection], fatigue, myalgia, and weight loss), lymphadenopathy, skin lesions (butterfly facial rash, discoid rash, urticaria, vasculitis, nonscarring alopecia, or undefined skin changes) [16], oral or nasopharyngeal ulcerations, photosensitivity, joints involvement (arthritis involving 2 or more joints, arthralgia), serositis (pleural effusion, pericardial effusion, pericarditis) [17], hematologic domain (leukopenia – white blood cell count < 4000/mm^3^ [18], anemia – male hemoglobin [Hb] ≤ 13.5 g/dl, female Hb ≤ 12 g/dl [19], hemolytic anemia – anemia with positive direct antiglobulin test and/or haptoglobin < 0.3 g/l [20], thrombocytopenia – platelet count < 100,000/mm^3^, macrophage activation syndrome (MAS) [21], thrombotic thrombocytopenic purpura (TTP) [22], kidney involvement (proteinuria > 0.5 g/day – after the exclusion of infection, pathological casts in urine sediment, erythrocyturia, leukocyturia – after the exclusion of infection) [23], nervous system involvement (symptoms of central or peripheral nervous involvement) according to American College of Rheumatology Nomenclature and Case Definitions for Neuropsychiatric Lupus Syndromes [24], Raynaud’s phenomenon, respiratory system involvement (interstitial lung disease [ILD], diffuse alveolar haemorrhage, pulmonary arterial hypertension [PAH], defined as a pulmonary artery systolic pressure > 45 mm Hg measured in transthoracic echocardiography) [25], lupoid hepatitis [26].

We also collected age at the LN diagnosis, histologic type of nephropathy (if kidney biopsy was performed), classified by the International Society of Nephrology/Renal Pathology Society (ISN/RPS) system, and data on end-stage renal disease (ESRD), if applicable [27]. LN was confirmed either by renal biopsy and classified according to the ISN/RPS criteria or based on overt renal symptoms (proteinuria, active urinary sediment) during a lupus flare [4]. ESRD was diagnosed when kidney transplantation or chronic dialysis was required due to no adequate kidney function in the long term [28].

We also analyzed the presence of internist comorbidities, such as arterial hypertension, diabetes mellitus, hypercholesterolemia, hypo- and hyperthyroidism, atrial fibrillation (AF), lower extremity peripheral artery disease (PAD), heart failure, malignancy, and any thromboembolic events, including stroke, transient ischemic attack (TIA), myocardial infarction (MI), deep vein thrombosis (DVT), pulmonary embolism. Arterial hypertension was defined as a history of blood pressure ≥ 140/90 mmHg or the preadmission of antihypertensive treatment. Diabetes mellitus was defined as a condition treated with insulin or oral hypoglycaemic agents or having a fasting serum glucose ≥ 7.0 mmol/l. Hypercholesterolemia was defined as previously diagnosed and treated with statins or having a serum low-density lipoprotein cholesterol ≥ 3.0 mmol/l. Hypothyroidism was defined as a previously diagnosed or clinical condition of thyroid hormone deficiency [29]. Hyperthyroidism was defined as a previously diagnosed or clinical condition of inappropriately high synthesis and secretion of thyroid hormones by the thyroid [30]. AF was defined as previously diagnosed or confirmed based on the results of an electrocardiography. Lower extremity PAD was defined as previously diagnosed or confirmed based on the resting ankle-brachial index (ABI) testing [31]. Heart failure (HF) was defined as previously diagnosed, similar to heart failure, or having left ventricular ejection fraction ≤ 40 mm Hg [32]. Malignancy was defined as previously diagnosed. In women, we also assessed the miscarriages. The treatment modalities included glucocorticoids, hydroxychloroquine or chloroquine, azathioprine, cyclosporine A, mycophenolate mofetil, cyclophosphamide, immunoglobulins intravenously in suppressive doses, biological agents (belimumab, rituximab, anifrolumab), currently or in the past. We also checked whether patients had plasmapheresis.

The Bioethics Committee of the Jagiellonian University Medical College has approved the research (No: N41/DBS/000936). All procedures adhered to the ethical principles outlined in the Declaration of Helsinki. Informed patient consent was waived due to a retrospective study design.

### Laboratory analysis

Laboratory data collected included the hematological, renal, and immunological parameters. Complete blood cell count (CBC), lipid profile, haptoglobin, creatinine with estimated glomerular filtration rate (eGFR, using Modification of Diet in Renal Disease formula), 24-hour urine protein excretion, urinary sediment analysis, direct antiglobulin test, and blood group designation were measured using routine laboratory techniques [33]. Anti-nuclear antibodies (ANA) were evaluated by indirect immunofluorescence (IIF) technique using Hep-2 cells. Anti-double-stranded DNA (anti-dsDNA) antibodies were assayed by IIF using Crithidia luciliae as substrate. Anti-SSA (Ro), anti-SSB (La), anti-histone, anti-nucleosome, anti-Smith (Sm), and anti-ribonucleoprotein (RNP) were identified by enzyme-linked immunosorbent assay (ELISA) or line-blot immunoassay (Euroline, Lϋbeck, Germany, all). Anti-myeloperoxidase (MPO) and anti-proteinase 3 (PR3) antibodies were assessed by a standardized ELISA technique. Serum complement levels (C3c and C4) and rheumatoid factor (RF) were assessed by nephelometry. In retrospective analysis, laboratory tests for hypercoagulability were also included, such as lupus anticoagulant (LA), anti-cardiolipin (aCL) and anti-beta-2-glycoprotein I (aβ2GPI) antibodies, both in IgM and IgG classes, antithrombin activity, protein C activity, free protein S level, activity of factor VIII, and presence of factor V Leiden and prothrombin G20210A gene variants. All of them were measured using routine laboratory techniques.

### Statistical elaboration

The results were analyzed using STATISTICA Tibco 13.3 software and the R software. Categorical variables were presented as frequencies (number of cases) with relative frequencies (percentages) and compared using the Chi^2^ test or the exact Fisher test. The normality of data distribution was evaluated using the Shapiro-Wilk test. All continuous variables were non-normally distributed and, thus, were presented as median with Q1-Q3 range and compared using the unpaired Mann-Whitney test. To calculate the odds ratio (OR) with a 95% confidence interval (CI), the cut-off points were calculated based on receiver operating characteristic (ROC) curves. A significance threshold of two-sided *p*-values < 0.05 was employed for all analyses.

## Results

### Patients characteristics

This study comprised 331 LN white Caucasian patients, constituting 35.94% of the total SLE patients (*n* = 921) treated in our center. As shown in Tables 1, 207 (62.54%) exhibited kidney manifestations within one year of the SLE diagnosis (early-onset LN). In comparison, 122 (36.86%) cases developed renal involvement later in the disease (delayed-onset LN). In 2 (0.6%) cases of LN the timing of the first kidney manifestation in the SLE course was unknown; consequently, these cases were not included in further analysis. The delayed-onset LN group was characterized by an almost two-fold higher female-to-male ratio and 4.5 years lower median age of SLE diagnosis than early-onset LN (26.5 vs. 31.0 years, respectively, *p* < 0.001), but with the same percentage of SLE diagnosed in childhood. Additionally, the median age at the diagnosis of LN was similar between early-onset and delayed-onset LN groups (31.0 vs. 33.0 years, respectively; *p* = 0.48). However, there was a significant difference in the median delay from SLE diagnosis to LN onset between the two groups. The second group experienced a longer delay (0.0 vs. 5.5 years, respectively; *p* < 0.001). Furthermore, the disease duration was notably longer in the delayed-onset LN, with a median of 18 years compared to 11 years in the early-onset group (*p* < 0.001), with no difference in the time delay from first symptoms to SLE diagnosis (Table 1).

**Table 1:** Demographic characteristics of 329 lupus nephritis patients

Characteristics	Early-onset LN patients *n* = 207	Delayed-onset LN patients *n* = 122	*p*-value
Age of SLE onset			
Adult onset (age of onset ≥ 18 years), n (%)	185 (89.4%)	101 (93.8%)	0.12
Juvenile onset (age of onset < 18 years), n (%)	22 (10.6%)	21 (6.2%)	
General characteristics			
Sex, female, n (%)	167 (80.3%)	109 (88.6%)	0.07
Female to male ratio	4.07	7.79	< 0.001*
Age at SLE diagnosis			
Median (Q1-Q3), years	31.0 (23.0–46.5)	26.5 (20.0–33.0)	< 0.001*
Age at LN diagnosis			
Median (Q1-Q3), years	31.0 (23.0–46.5)	33.0 (26.0–44.0)	0.48
Age at first SLE symptoms			
Median (Q1-Q3), years	30.0 (22.0–45.0)	24.0 (19.0–33.0)	< 0.001*
Time delay between onset of SLE symptoms and diagnosis			
Median (Q1-Q3), months	0 (0–12)	0 (0–12)	1.00
Age at last visit			
Median (Q1-Q3), years	45.0 (34.0–59.0)	43.0 (36.0–53.0)	0.99
Disease duration			
Median (Q1-Q3), years	11.0 (5.0–20.0)	18.0 (12.3–24.0)	< 0.001*

Table 1 footnotes. Categorical variables are presented as numbers with percentages, and continuous variables are presented as medians with Q1 and Q3 ranges. The statistically significant results are marked with an asterisk. Abbreviations: LN – lupus nephritis, *n* – number

Among the LN patients, 92 individuals (27.96%) had a positive family history of autoimmune diseases; in 13 cases, more than one family member was affected by an autoimmune disease. The most frequently recorded conditions were rheumatoid arthritis (*n* = 40, 12.08% overall), SLE (*n* = 20, 6.04% overall), and psoriasis (*n* = 9, 2.72% overall). Other sporadically observed conditions included systemic sclerosis (*n* = 1, 0.3% overall), granulomatosis with polyangiitis (*n* = 1, 0.3% overall), mixed connective tissue disease (*n* = 1, 0.3% overall), Sjögren’s syndrome (*n* = 1, 0.3% overall), ulcerative colitis (*n* = 1, 0.3% overall), and arthritis of other etiology (*n* = 19, 5.74% overall). However, no significant differences were found in the comparison between the two studied subgroups (*p* > 0.05, all).

Also, the frequency of internist comorbidities was similar in both subgroups, including arterial hypertension, diabetes mellitus, HF, hypercholesterolemia, and PAD. Additionally, analyzed subgroups did not differ in ESRD, monoclonal gammopathy of undetermined significance, malignancy, and thrombotic or miscarriage rates. Detailed information is presented in Table 2. Heart failure (17.39% vs. 6.19%; *p* = 0.042), atrial fibrillation (13.04% vs. 3.58%; *p* = 0.03), pleural effusion (52.17% vs. 25.66%; *p* = 0.006), anemia (100% vs. 83.28%; *p* = 0.033), particularly hemolytic anemia (70% vs. 27.34%; *p* = 0.005), thrombocytopenia (60.87% vs. 32.11%; *p* = 0.005), and pulmonary artery hypertension (13.04% vs. 2.02%; *p* = 0.002) co-existed independently with ESRD. Furthermore, ESRD cases also had higher odds ratios for therapy with immunoglobulins in suppressive doses (13.76, 95% CI: 3.84–48.127, *p* < 0.001) and plasmapheresis (7.74, 95% CI: 2.52–22.57, *p* < 0.001) in medical history, as well as death risk (6.14, 95% CI: 4.02–62.13, *p* < 0.001).

**Table 2:** Cumulative frequencies of comorbidities in lupus nephritis patients

Comorbidities^a^	Early-onset LN patients *n* = 207	Delayed-onset LN patients *n* = 122	*p*-value
Hypothyroidism, *n* (%)	49 (23.56%)	28 (22.76%)	0.96
Hyperthyroidism, *n* (%)	13 (6.25%)	3 (2.44%)	0.19
Arterial hypertension, *n* (%)	154 (74.04%)	87 (70.73%)	0.60
Diabetes mellitus, *n* (%)	24 (11.54%)	14 (11.38%)	0.89
Hearth failure^b^, *n* (%)	13 (6.25%)	10 (8.13%)	0.67
Hypercholesterolemia^c^, *n* (%)	143 (69.08%)	80 (65.04%)	0.52
Atrial fibrillation, *n* (%)	10 (4.81%)	4 (3.25%)	0.69
Peripheral artery disease, *n* (%)	7 (3.37%)	4 (3.28%)	0.78
End-stage renal disease, *n* (%)	15 (7.21%)	8 (6.56%)	0.99
Monoclonal gammopathy of undetermined significance, *n* (%)	8 (3.85%)	1 (0.81%)	0.20
Malignant tumor, *n* (%)	20 (9.62%)	11 (8.94%)	0.99
Artery thrombotic episode, *n* (%)	58 (27.88%)	42 (34.15%)	0.28
Stroke, *n* (%)	13 (6.25%)	8 (6.5%)	0.89
Transient ischemic attack, *n* (%)	3 (1.44%)	3 (2.44%)	0.82
Myocardial infarct, *n* (%)	52 (25%)	36 (29.27%)	0.47
Thrombotic episode in another artery, *n* (%)	4 (1.92%)	3 (2.44%)	0.94
Venous thrombotic episode, *n* (%)	32 (15%)	29 (24%)	0.09
Deep venous thrombosis, *n* (%)	25 (12%)	25 (20%)	0.06
Pulmonary embolism, *n* (%)	10 (5%)	4 (3%)	0.69
Deep venous thrombosis and pulmonary embolism, *n* (%)	5 (2%)	3 (2%)	0.73
Thrombotic episode in another venous, *n* (%)	4 (2%)	4 (3%)	0.70
Miscarriage^d^, *n* (%)	15 (10.14%)	17 (17.53%)	0.07

Table 2 footnotes. ^a^ – comorbidities actually or in the past, ^b^ – symptoms of heart failure or EF ≤ 40% or diagnosis based on medical history, ^c^ – LDL > 3 mmol/l or pharmacotherapy with statin or diagnosis based on medical history, ^d^ – % of women with miscarriage from a number of women with systemic lupus erythematosus. Abbreviations: LDL – low-density lipoprotein, LN – lupus nephritis, *n* – number

### Patients with delayed-onset lupus nephritis presented more severe clinical manifestations

Then, we analyzed SLE clinical characteristics in both LN subgroups. Interestingly, we documented that the delayed-onset LN had more severe clinical manifestations other than kidney involvement, including hematological signs (97.56%), joint inflammation (91.87%), and constitutional symptoms (86.18%) (Table 3). Early-onset lupus nephritis (LN) patients exhibited similar predominant symptoms, but joint involvement was less frequent in this LN group, with corresponding percentages of 93.75%, 79.81%, and 77.88%, respectively, for the three manifestations (*p* = 0.19, *p* = 0.003, *p* = 0.09). Furthermore, delayed-onset LN patients had more common other clinical SLE manifestations, such as mucocutaneous (∼ 1.15 times, *p* = 0.01), lymphadenopathy (∼ 1.83 times, *p* = 0.009), Raynaud’s phenomenon (∼ 1.63 times, *p* = 0.047), and myalgias (∼ 1.55 times, *p* = 0.002). Interestingly, there were no significant differences in serositis, hemolytic anemia, lymphopenia, thrombocytopenia), lupoid hepatitis, neurological and lung abnormalities frequency between analyzed LN groups (Table 3). Additionally, the results of antithrombin activity, protein C activity, free protein S level, factor VIII activity, and prevalence of factor V Leiden and prothrombin G20210A gene variants were similar in both subgroups (data not shown). Moreover, the blood ABO groups and Rh blood types were similar between both LN groups (*p* > 0.05, data not shown).

**Table 3:** Cumulative frequencies of systemic involvement in lupus nephritis cohort

Clinical manifestations	Early-onset LN patients *n* = 207	Delayed-onset LN patients *n* = 122	*p*-value
Constitutional manifestations, *n* (%)	162 (77.88%)	106 (86.18%)	0.09
Fever, *n* (%)	94 (48.7%)	72 (59.5%)	0.08
Fatigue/weakness, *n* (%)	129 (65.82%)	93 (76.86%)	0.05
Myalgias, *n* (%)	62 (31.79%)	60 (49.18%)	0.002*
Weight loss, *n* (%)	51 (26.42%)	30 (24.79%)	0.85
Lymphadenopathy, *n* (%)	30 (15.38%)	34 (28.1%)	0.009*
Mucocutaneus manifestations, *n* (%)	146 (70.19%)	102 (80.93%)	0.01*
Butterfly facial rash, *n* (%)	68 (33.33%)	62 (50.82%)	0.003*
Discoid rash, *n* (%)	10 (4.93%)	12 (9.84%)	0.14
Urticaria, *n* (%)	9 (4.43%)	17 (13.93%)	0.004*
Cutaneous vasculitis, *n* (%)	9 (4.43%)	14 (11.48%)	0.03*
Alopecia, *n* (%)	40 (19.61%)	45 (36.89%)	< 0.001*
Oral and/or nasal ulcers, *n* (%)	26 (12.81%)	25 (20.49%)	0.09
Photosensitivity, *n* (%)	46 (22.66%)	43 (35.25%)	0.02*
Other skin changes, *n* (%)	118 (57.28%)	88 (71.54%)	0.01*
Joint manifestations, *n* (%)	166 (79.81%)	113 (91.87%)	0.003*
Arthritis, *n* (%)	105 (51.72%)	89 (72.95%)	< 0.001*
Arthralgia, *n* (%)	165 (80.1%)	114 (92.68%)	0.004*
Serositis, *n* (%)	71 (34.3%)	51 (41.8%)	0.21
Pleural effusion, *n* (%)	52 (25.12%)	38 (31.4%)	0.27
Pericardial effusion, *n* (%)	42 (20.69%)	33 (28.21%)	0.16
Pericarditis, *n* (%)	5 (2.42%)	7 (5.74%)	0.21
Hematological manifestations, *n* (%)	195 (93.75%)	120 (97.56%)	0.19
Leucopenia^a^, *n* (%)	117 (58.21%)	92 (76.67%)	0.001*
Lymphopenia^b^, *n* (%)	157 (81.77%)	104 (86.67%)	0.33
Anemia^c^, *n* (%)	169 (84.5%)	103 (84.43%)	0.89
Hemolytic anemia^d^, *n* (%)	31 (36.05%)	11 (21.15%)	0.19
Thrombocytopenia^e^, *n* (%)	68 (34%)	42 (34.43%)	0.97
Positive direct Coombs test, *n* (%)	17 (32.69%)	12 (42.86%)	0.51
Macrophage activation syndrome, *n* (%)	5 (2.44%)	0 (0%)	0.20
Thrombotic thrombocytopenic purpura, *n* (%)	1 (0.48%)	0 (0%)	0.63
Kidney involvement, *n* (%)	207 (100%)	122 (100%)	1.00
24-hour urinary protein excretion > 0.5 g/day, *n* (%)	188 (95.92%)	112 (97.39%)	0.72
24-hour urinary protein excretion > 3.5 g/day, *n* (%)	92 (56.79%)	66 (61.68%)	0.50
Urinary casts, *n* (%)	75 (55.56%)	63 (69.23%)	0.05
Erythrocyturia, *n* (%)	141 (84.94%)	88 (84.62%)	0.53
Leukocyturia, *n* (%)	144 (82.76%)	98 (87.5%)	0.36
Neurological abnormality, *n* (%)	36 (17.31%)	23 (18.7%)	0.86
Central nervous system involvement, *n* (%)	28 (13.59%)	16 (13.01%)	0.99
Peripheral nervous system involvement, *n* (%)	13 (6.31%)	11 (8.94%)	0.50
Raynaud’s phenomenon, *n* (%)	31 (14.98%)	30 (24.39%)	0.047*
Lung involvement, *n* (%)	17 (8.17%)	13 (10.57%)	0.59
Interstitial lung disease, *n* (%)	10 (4.83%)	9 (7.32%)	0.49
Diffuse alveolar heamorrhage, *n* (%)	3 (1.45%)	5 (4.07%)	0.26
Pulmonary hypertension, *n* (%)	7 (3.47%)	2 (1.68%)	0.56
Lupoid hepatitis, *n* (%)	9 (4.35%)	4 (3.25%)	0.84

Table 3 footnotes. Categorical variables are presented as numbers with percentages. The statistically significant results are marked with an asterisk. Abbreviations: *n* – number, lupus nephritis – LN, ^a^ – < 4000/mm^3^ or diagnosed in medical history, ^b^ – < 1500/mm^3^ or diagnosis based on medical history, ^c^ – ≤ 12 g/dl in women, ≤ 13.5 g/dl in men, or diagnosis based on medical history, ^d^ – anemia with a positive direct Coombs test or anemia with a decreased level of haptoglobin or diagnosis based on medical history, ^e^ – <100,000/mm^3^ or diagnosis based on medical history

Throughout the median follow-up period of 14 years, a total of 16 (5.28%) LN patients died, with 12 cases (6.35%) in the early-onset LN group and 4 (3.51%) cases in the delayed-onset LN group (*p* = 0.28). The most common causes of death were infection (6 cases, 37.50%), SLE exacerbation (3 cases, 18.75%), and malignancies (2 cases, 12.5%), with no differences between the analyzed subgroups (*p* > 0.05 for all).

### Autoantibodies profile in both lupus nephritis subgroups in the course of systemic lupus erythematosus

All recruited patients had ANA in the titer of above 1:160, and anti-dsDNA antibodies were the most prevalent in both subgroups (Table 4). However, in delayed-onset LN, we documented more frequent anti-dsDNA and anti-Smith antibodies, with 1.92 (95% CI: 1.29–2.85, *p* = 0.001) and 1.52 (95% CI: 1.09–2.12, *p* = 0.013) higher OR, respectively, than in the early-onset LN subgroup. The former subgroup also had a higher prevalence of anti-Ro (60% vs. 42.84%, *p* = 0.004) and anti-RNP (39.17% vs. 15.38%, *p* < 0.001) antibodies. Anti-PR3 antibodies were examined in 77 (23.26%) out of 331 LN patients, and anti-MPO antibodies were assessed in 80 (24.17%) out of 331 LN patients. Anti-PR3 antibodies tested positive in only 5 (1.51% of all LN patients), whereas anti-MPO antibodies were positive in 8 (2.42% of all LN patients) without statistically significant differences between LN groups (*p* > 0.05 for both). Concerning antiphospholipid antibodies, there was a higher incidence of aCL antibodies in the IgG class among delayed-onset LN patients. Furthermore, 17 (5.17%) cases of early-onset LN and 13 (3.95%) cases of late-onset LN exhibited triple positivity for antiphospholipid syndrome, with no statistically significant differences between the two groups (*p* = 0.54). Renal biopsy was performed only in 17 (56.67%) of these triple-positive patients, and none showed signs of antiphospholipid nephropathy. Additional information regarding the presence of specific autoantibodies in the LN cohort in the course of SLE is provided in Table 4.

**Table 4:** Laboratory findings in lupus nephritis patients in the course of SLE.

Laboratory parameter (number of patients with analyzed parameter)	Early-onset LN patients *n* = 207	Delayed-onset LN patients *n* = 122	*p*-value
Rheumatoid factor, *n* (%)	14 (15.56%)	19 (27.94%)	0.17
ANA – IIF assay			
Anti-SSA/Ro antibodies^a^, *n* (%)	84 (42.86%)	72 (60.0%)	0.004*
Anti-SSB/La antibodies^a^, *n* (%)	41 (20.92%)	31 (25.83%)	0.38
Anti-histone antibodies^a^, *n* (%)	68 (34.69%)	51 (42.5%)	0.20
Anti-nucleosome antibodies^a^, *n* (%)	85 (43.37%)	60 (50.0%)	0.30
Anti-Smith antibodies^a^, *n* (%)	18 (9.28%)	23 (19.17%)	0.02*
Anti-RNP antibodies^a^, *n* (%)	30 (15.38%)	47 (39.17%)	< 0.001*
Anti-dsDNA antibodies^a^, *n* (%)	94 (47.96%)	69 (57.5%)	0.13
Anti-dsDNA antibodies^b^, *n* (%)	155 (79.08%)	111 (93.28%)	0.001*
Antineutrophil cytoplasmic antibodies			
Anti-PR3 antibodies^c^, *n* (%)	3 (6.52%)	2 (6.45%)	0.65
Anti-MPO antibodies^c^, *n* (%)	3 (6.12%)	6 (18.75%)	0.16
Antiphospholipid antibodies			
Lupus anticoagulant, *n* (%)	36 (23.23%)	28 (29.47%)	0.34
Anti-cardiolipin antibodies IgG and/or IgM, *n* (%)	85 (51.83%)	65 (61.32%)	0.10
Anti-cardiolipin antibodies IgG, *n* (%)	64 (39.26%)	56 (53.33%)	0.03*
Anti-cardiolipin antibodies IgM, *n* (%)	55 (33.74%)	36 (34.62%)	0.99
Anti-β2 glycoprotein I IgG and/or IgM, *n* (%)	29 (20.71%)	17 (21.25%)	0.94
Anti-β2 glycoprotein I IgG, *n* (%)	18 (13.14%)	11 (14.47%)	0.95
Anti-β2 glycoprotein I IgM, *n* (%)	17 (12.5%)	10 (12.99%)	0.91

Table 4 footnotes. ^a^ – immunoblotting assay, ^b^ – CLIFT (the *Crithidia luciliae* immunofluorescence test), ^c^ – ELISA (Enzyme-Linked Immunosorbent Assay). The statistically significant results are marked with an asterisk. Abbreviations: *n* – number, lupus nephritis – LN, ANA – anti-nuclear antibodies, dsDNA – double-stranded DNA, IIF – indirect immunofluorescence, MPO – myeloperoxidase, PR3 – proteinase 3, RNP – ribonucleoprotein

### Histopathological examination in lupus nephritis patients

Renal biopsies were performed in 180 cases, representing 54.71% of LN patients. In others, that procedure was not completed due to no patient consent, renal anatomical abnormalities, presence of one functional kidney, pregnancy, bleeding diathesis, uncontrolled arterial hypertension, and general severe condition of the patient. Overall, in LN patients the most common histological type was class IV (diffuse proliferative glomerulonephritis), identified in 91 cases (50.56%), followed by classes II (*n* = 33, 18.33%), III (*n* = 26, 14.44%), V (*n* = 22, 12.22%), and VI (*n* = 5, 2.78%), with class I detected in only 3 patients (1.67%). Interestingly, both LN groups had similar LN class frequencies according to this classification, and the most often stated type was the class IV revealed in 46.02% early-onset LN (*n* = 52) and 56.52% delayed-onset LN (*n* = 39). Interestingly, class IV was characterized by higher urinary protein excretion, particularly with values exceeding 3.5 g/day, more aggressive therapy mode, including the administration of cyclophosphamide, and positive results of direct antiglobulin test (Table 5). Moreover, confirmation of non-immune-complex-mediated disease was established in 5 (2.78%) patients.

**Table 5:** Features in lupus nephritis patients with performed renal biopsy; histological classes according to International Society of Nephrology/Renal Pathology Society classification

Feature	Renal biopsy histological classes according to ISN/RPS	*n* (%) positive results	*p*-value
Cyclophosphamide treatment			
	I, *n* (%)	1 (0.57%)	< 0.001*
	II, *n* (%)	15 (8.52%)	
	III, *n* (%)	18 (10.23%)	
	IV, *n* (%)	77 (43.75%)	
	V, *n* (%)	18 (10.23%)	
	VI, *n* (%)	5 (2.84%)	
	Total, *n* (%)	134 (76.14%)	
Positive direct antiglobulin test			
	I, *n* (%)	2 (3.85%)	0.03*
	II, *n* (%)	2 (3.85%)	
	III, *n* (%)	5 (9.62%)	
	IV, *n* (%)	6 (11.54%)	
	V, *n* (%)	1 (1.92%)	
	VI, *n* (%)	1 (1.92%)	
	Total, *n* (%)	17 (32.69%)	
24-hour urinary protein excretion > 3.5 g/day			
	I, *n* (%)	1 (0.67%)	< 0.001*
	II, *n* (%)	9 (6.04%)	
	III, *n* (%)	8 (5.37%)	
	IV, *n* (%)	61 (40.94%)	
	V, *n* (%)	19 (12.75%)	
	VI, *n* (%)	4 (2.68%)	
	Total, *n* (%)	102 (68.46%)	

Table 5 footnotes. The statistically significant results are marked with an asterisk. Abbreviations: ISN/RPS – International Society of Nephrology/Renal Pathology Society, *n* – number

### More aggressive immunosuppressive treatment in delayed-onset lupus nephritis

Next, we investigated different immunosuppressive treatment modalities. Data on this topic is provided in Table 6. Among delayed- and early-onset LN patients, the most frequently were administered steroids (100% vs. 99.03%, respectively, *p* = 0.25). In turn, delayed-onset LN patients were prescribed more often with other immunosuppressant regimens, such as mycophenolate mofetil, belimumab, and azathioprine, which were used 1.27-times, 2.87-times, and 1.55-times, respectively, more prevalent in them (*p* < 0.01, all). Patients with ESRD received more often immunoglobulins and plasmapheresis (*p* < 0.001, for both), while those who died mycophenolate mofetil and immunoglobulins (*p* = 0.003 and *p* = 0.04, respectively).

**Table 6:** Treatment patterns in lupus nephritis patients

Treatment	Early-onset LN patients *n* = 207	Delayed-onset LN patients *n* = 122	*p*-value
Glucocorticoids oral and/or intravenous, *n* (%)	204 (99.03%)	123 (100.0%)	0.25
Chloroquine or hydroxychloroquine, *n* (%)	117 (57.64%)	99 (81.15%)	< 0.001*
Azathioprine, *n* (%)	86 (42.36%)	80 (65.57%)	< 0.001*
Cyclosporine, *n* (%)	21 (10.4%)	17 (13.93%)	0.39
Belimumab, *n* (%)	7 (3.48%)	12 (10.0%)	0.007*
Mycophenolate mofetil, *n* (%)	119 (59.2%)	91 (75.2%)	0.003*
Cyclophosphamide, *n* (%)	129 (63.9%)	80 (65.6%)	0.71
Rituximab, *n* (%)	12 (5.97%)	9 (7.5%)	0.79
Immunoglobulins, *n* (%)	7 (3.48%)	10 (8.26%)	0.04*
Plasmapheresis, *n* (%)	16 (7.96%)	11 (9.17%)	0.75
Anifrolumab, *n* (%)	1 (0.5%)	3 (2.5%)	0.11

Table 6 footnotes. The statistically significant results are marked with an asterisk. Abbreviations: *n* – number, lupus nephritis – LN

## Discussion

The present study provides valuable insights into the demographic, clinical, and laboratory characteristics of the large cohort of 329 white Polish early-onset and delayed onset LN patients. The findings shed light on several key aspects of this condition, with potential implications for developing diagnostic and therapeutic strategies. To our knowledge, this is one of the largest LN groups described in the literature.

More patients had early-onset LN, similar to the study conducted by Delfino et al. [34]. In turn, delayed-onset was characterized by a significantly higher female-to-male ratio and younger age at the SLE diagnosis than early-onset patients. In a study on LN in three groups according to the age of diagnosis, Shabaka et al. [35] reported a high female-to-male ratio in all three analyzed cohorts (pediatric onset [< 18 years old], adult-onset [18–50 years old], delayed-onset [> 50 years old]). Interestingly, according to a study by Trentin et al. [36], the prevalences of renal disease between males and females with SLE were similar. Therefore, distinctions in sex in LN highlight the possibility of different underlying factors contributing to the disease’s onset in these age-related groups, prompting further exploration into genetic and hormonal factors.

Interestingly, the presence of a family history of systemic autoimmune diseases was comparable between both analyzed subgroups. This data is novel since detailed information on family history considering specific autoimmune disorders is lacking in current literature.

Next, delayed-onset LN patients exhibited more severe clinical manifestations compared to their early-onset counterparts, including a higher occurrence of joint and mucocutaneous signs, lymphadenopathy, Raynaud’s phenomenon, myalgias, and leucopenia. This finding highlights the importance of early LN recognition in SLE patients with no kidney involvement so far, as well as aggressive therapy while being diagnosed, to mitigate severe outcomes. Moreover, delayed-onset LN patients exhibited a higher frequency of autoantibodies, including anti-dsDNA and anti-Sm, suggesting a more robust autoimmune response.

The histopathological classification of LN revealed that class IV (diffuse proliferative glomerulonephritis) was the most common, consistent with current literature [37]. Importantly, this classification was associated with a higher urinary protein excretion, more frequent use of cyclophosphamide, and positive direct Coomb’s test. That suggests that the ISN/RPS classification remains a valuable tool for assessing disease severity and guiding treatment decisions, which might be helpful as a predictive tool. Lately, in a study on children by Das et al. [38], class IV LN was associated with proteinuria, hematuria, and positivity for ANA and anti-dsDNA antibodies. Additionally, a similar conclusion was drawn from the different study on patients > 16 years old by Silaide de Araújo Júnior et al. [39]; this class was related to the positive anti-dsDNA antibodies. Therefore, the described biomarkers might be helpful in clinical care in predicting the histopathological type of kidney injury, especially when a kidney biopsy is unavailable or contraindicated for medical reasons.

Our study did not demonstrate differences in anti-PR3 and anti-MPO antibody prevalence between analyzed LN subgroups. ANCA might be called the more aggressive nephritic and nephrotic syndrome [40]; however, we did not observe such an association in our cohort. This discrepancy can be associated with the interference of ANA in the differentiation between pANCA and cANCA patterns [41] and a small study group with checked ANCA antibodies by ELISA method and not by indirect immunofluorescence in our research.

In contrast to our results, a study by Kang et al. [42] showed that poor renal outcomes occurred in almost 30% of analyzed patients with non-proliferative LN. Moreover, several factors have been linked to unfavorable kidney function, including older age, lower eGFR, or failure to reach complete remission. Indeed, delayed-onset LN is perceived to be a risk factor for the relapse of LN [43]. Yet, no renal remission is a risk factor for chronic kidney disease and ESRD [44]. Recently, Tang et al. [45] suggested that erythrocyte sedimentation rate (ESR), mucosal ulcers, proteinuria, and hematuria are independent risk factors for LN in SLE patients as a valuable hint in clinical work. Obviously, proteinuria and hematuria were independent risks of LN, whereas the presence of oral and/or nasal ulcers was not related to renal manifestation in our SLE cohort (data not shown). Interestingly, a paper by Hsu et al. [46] concluded that among risk factors for LN in juvenile SLE patients were the occurrence of high anti-dsDNA antibody and ESR levels during the follow-up period. Additionally, Du et al. [47] found that female sex with shorter disease duration, partial response within six months of therapy, and lower urine protein/creatinine ratio were markers of achieving complete renal remission during treatment. However, a recent study by Duran et al. [48] showed that complete and partial renal responses were similar between proliferative and non-proliferative LN patients in a 2-year follow-up analysis. Moreover, in the latest study on early-onset and delayed-onset lupus nephritis by Cho et al. [49], ESRD was found to be associated with aggressive immunosuppressive therapy, aligning with our findings. Next, the risk of ESRD was comparable between early-onset LN and delayed-onset LN cases, which also stays in line with our results.

Interestingly, the study did not find significant differences in the prevalence of internist comorbidities between early-onset and delayed-onset LN patients. One may say that the presence of LN itself may be a significant risk factor for these comorbid conditions, irrespective of the time of LN diagnosis. Next, we reported no differences in the causes of death between early-onset and delayed-onset LN patients, with infections and SLE exacerbations as the most common causes of death. That highlights the need for vigilant infection prevention and aggressive SLE management in both subgroups. The study also revealed differences in immunosuppressive treatment between early-onset and delayed-onset LN patients. Delayed-onset LN patients with more severe disease forms were more likely to receive immunosuppressive regimens, such as azathioprine, mycophenolate mofetil, and belimumab. Interestingly, recent EULAR recommendations suggest that active LN patients might benefit apart from immunosuppressive treatment, including glucocorticosteroids, mycophenolate mofetil or cyclophosphamide, also from the therapy with belimumab or voclosporin/tacrolimus [50].

### Study limitation

First, the study’s retrospective nature may introduce inherent biases in data collection. Next, single-center design may limit the generalizability of the results to a broader population, as regional variations in disease characteristics and management practices could exist. Additionally, we included adult-onset and juvenile-onset LN patients. Since they have different disease courses, it may impact the presented results. Finally, the absence of a control group of non-SLE individuals or SLE patients without LN may limit the ability to draw definitive conclusions about the factors specific to LN development and outcomes.

## Conclusions

In conclusion, the current study provides valuable insights into the demographic, clinical, and laboratory characteristics of LN in a Polish population. It highlights the distinct features of delayed-onset LN, including a higher female predominance and more severe clinical manifestations. The study reaffirms the importance of the ISN/RPS histopathological classification system in assessing disease severity. Furthermore, it emphasizes the need for aggressive immunosuppressive therapies in LN patients and the potential role of autoantibodies as biomarkers for renal involvement. Prospective studies with larger and more diverse cohorts are needed to validate the findings and inform the development of tailored diagnostic and therapeutic strategies for LN patients.

## Data Availability

The data presented in this study are available upon reasonable request from the corresponding author.
